# Total neoadjuvant chemotherapy with FLOT scheme in resectable adenocarcinoma of the gastro-oesophageal junction or gastric adenocarcinoma: impact on pathological complete response and safety

**DOI:** 10.3332/ecancer.2021.1168

**Published:** 2021-01-13

**Authors:** Luis Villanueva, Jaime Anabalon, Jean M Butte, Pamela Salman, Sergio Panay, Elizabeth Milla, Carlos Gallardo, Sebastian Hoefler, Roberto Charles, Felipe Reyes, Olga Barajas, Luis Matamala, Angelica Molina, Sergio Portiño, Marcela Berrios, Christian Caglevic, Mauricio Mahave

**Affiliations:** 1 Department of Oncology, Instituto Oncologico Fundacion Arturo Lopez Perez, Santiago, 7500921, Chile; 2 Department of Oncology, Hospital Clinico Universidad de Chile, Santiago, 8380456, Chile; 3 Department of Oncology, Hospital Clinico San Borja Arriaran, Santiago, 8360160, Chile; 4 Department of Oncology, Instituto Nacional del Cancer, Santiago, 8380455, Chile

**Keywords:** total neoadjuvant chemotherapy, preoperative chemotherapy, FLOT, gastric cancer, adenocarcinoma of the gastro-oesophageal junction, pathological response

## Abstract

**Background:**

Gastric cancer is the fifth cause of cancer incidence worldwide. Multidisciplinary approaches that improve the survival are needed. Perioperative chemotherapies show improvement in pathological complete remission (pCR) and overall survival (OS), but less than 50% of the patients completed the chemotherapeutic regimen. The recent 5-fluorouracil, leucovorin, oxaliplatin, docetaxel-4 (FLOT4) study shows OS 50 months and pCR 16.6%, but only 46% of the patients completed pre- and postoperative treatment. This case series report evaluated pCR and safety in patients that received complete preoperative chemotherapeutic with FLOT.

**Methods:**

Patients received eight cycles FLOT regimen before surgery. Each cycle comprised 50 mg/m^2^ docetaxel intravenous (iv) on day 1, 85 mg/m^2^ oxaliplatin iv on day 1, 200 mg/m^2^ leucovorin iv on day 1 and 2,600 mg/m^2^ 5-fluorouracil iv in a 24-hour infusion on day 1, every 2 weeks.

**Results:**

Fifty-nine patients were evaluated, 58 patients received preoperative cycles. Thirty-one patients received all eight cycles of preoperative therapy. 65.5% patients presented any major adverse event. Thirty-nine patients underwent surgery. Thirty-three biopsy reports were obtained. Six patients (18.2%) presented pCR, 13 patients (39.4%) had no lymph node involvement. OS was 21.32 months. Patients with histology of signet ring carcinoma cells had a shorter survival than other histologies.

**Conclusion:**

Total neoadjuvant with FLOT chemotherapy presents an adequate safety profile, a similar pathologic regression rate, and a slightly higher rate of completing treatment to report in perioperative FLOT regimen studies. A prospective clinical study with suitable diagnostic, staging tools and an adequate follow-up may prove total neoadjuvant chemotherapy’s efficacy.

## Introduction

Gastric cancer is the fifth cause of cancer incidence worldwide and the third cause of global mortality, with 1,033,701 new cases and 782,685 deaths in 2018, suggesting this cancer has a high mortality rate. Chile has one of the highest age standardised incidence rates in the world (17.8 cases per 100,000 people), surpassed only by Asian countries, and a high age standardised mortality rate (11.5 deaths per 100,000 people) when compared to the rest of the countries of the Western world [1]. There were 3,250 deaths in 2016 [2]. Multidisciplinary approaches that improve the survival of patients with gastric cancer are needed. Among them, perioperative chemotherapies seem to help in controlling the disease.

Studies evaluating perioperative chemotherapies, such as the MAGIC study (Medical Research Counsil Adjuvant Gastric Infusional Chemotherapy), in which three cycles of epirubicin, cisplatin and fluorouracil or capecitabine (ECF/ECX) were indicated before and after surgery, showed an improvement in the 5-year survival rate when compared to surgery alone, 36% versus 23% (*p* = 0.009), and good rates of pathologic remission after chemotherapy treatment, although only 41.6% of the patients can complete the six cycles planned [3]. In the FNCLCC/FFCD 9703 study, patients received two or three preoperative cycles of cisplatin and fluorouracil (FU) and three or four postoperative cycles of the same scheme, improving the 5-year survival rate when compared to patients underwent surgery alone, 38% versus 24% (*p* = 0.02), but only 22.9% of the patients completed four postoperative cycles [4]. The recent FU, leucovorin, oxaliplatin and docetaxel-4 (FLOT4) study, which uses four cycles of FLOT before and after surgery, showed a median survival of 50 months versus 35 months with the ECF/ECX scheme [5]. The complete response rate of this scheme was 16.6% [6], but only 46% of the patients completed the full pre- and postoperative treatment.

These studies show that better disease-free survival and overall survival (OS) are related to a better pathologic complete remission, and higher rates of complete remission are obtained when the number of administered chemotherapy cycles is increased, e.g. 17% of pathologic complete remission after four cycles and 20% after six cycles [7, 8].

This retrospective study evaluated the safety and the rate of pathologic complete remission of complete preoperative chemotherapy with FLOT scheme.

## Methods

### Study design

This trial is a retrospective study, case series report, performed at the Instituto Oncologico Fundación Arturo López Pérez (FALP), Santiago, Chile. The study was carried out according to the local institutional guidelines and was approved by the local committee. The author holds responsibility for the collection and evaluation of data.

The primary objective of the study was to determine the rate of pathological remission and the safety of patients who underwent a total neoadjuvant regimen with FLOT regimen before surgery. Other objectives were intensity of treatment received, type and complications of surgery, OS of all patients treated with preoperative chemotherapy and according histological subtype.

All patients were presented to the cancer multidisciplinary team meeting of experts in the treatment of gastric cancer who prescribed total neoadjuvant therapy with FLOT regimen between November 2016 and May 2019.

Eligible patients had gastric or gastro-oesophageal junction cancer histologically confirmed as adenocarcinomas in clinical stage T1–T4 with involvement lymph node (N+), or extensive T3N0, or T4N0. The tumours were classified according to the Tumour-Node-Metastasis (TNM) Classification of Malignant Tumours of the Union for International Cancer Control (UICC), 7th edition. The clinical stage was assigned by evaluating the physical exam, an oesophagus-stomach-duodenum endoscopy and computerised tomography scans of the thorax, abdomen and pelvis.

### Procedures

Patients received eight cycles of FLOT chemotherapeutic regimen administered before surgery. Each cycle, performed every 2 weeks, comprised 50 mg/m^2^ docetaxel intravenous (iv) on day 1, 85 mg/m^2^ oxaliplatin iv on day 1, 200 mg/m^2^ leucovorin iv on day 1 and 2,600 mg/m^2^ 5-FU iv in a 24-hour infusion on day 1. The doses of treatment could reduce and/or be temporarily or permanently suspended due to unacceptable toxicity, disease progression or at the physicians’ discretion.

Surgery was programmed 4 weeks after the last dose of chemotherapy. Patients underwent a gastrectomy with transhiatal distal oesophagectomy and a D2 lymphadenectomy at our institution FALP.

Patients were evaluated according to age at the time of diagnosis, histological type, body mass index (BMI), the Eastern Cooperative Oncology Group (ECOG) Scale of Performance Status, haemoglobin levels, albumin levels, number of administered cycles, dose reductions, adverse reactions, type of surgery, surgical mortality during the first 30 days, anatomopathological results after treatment and OS.

Serious adverse events were defined as any adverse events of grade 3, 4 or 5, according to the National Cancer Institute Common Terminology Criteria for Adverse Events version 3.0.

### Pathological assessment

Archived haematoxylin and eosin-stained slides were evaluated by pathologists for accuracy of diagnosis. Formalin-fixed and paraffin-embedded tissue blocks were selected for tissue slides for immunohistochemistry. Cores of 5 mm with representative invasive tumour were analysed.

Histopathological reports included diagnosis according to World Health Organization (WHO) classification, UICC stage, presence or absence of lymphatic, vascular and perineural invasion and assessment of oral and distal resection margin and nodal involvement.

The tumours were classified adenocarcinomas: signet ring, tubular, mucinous, papillary, uncommon variants and not otherwise specified (NOS)

### Statistics

GraphPad Prism version 8.0 was used for all statistical calculations. For exploratory and descriptive analysis, scale variables were determined as median (range) and ordinal or dichotomous variables as absolute and relative frequencies. Survival data was plotted and analysed according to the Kaplan–Meyer method. For statistical testing, the following methods were used: Log-rank (Mantel–Cox) test and Hazard Ratio (logrank).

The overall survival was determined as period of time from first chemotherapy to death. The dates of death were recollected by death records provided by the civil registration national office until 22 September 2019.

## Results

Fifty-nine patients received the indication of total neoadjuvant chemotherapy with FLOT between November 2016 and May 2019. Patients were evaluated until 22 September 2019.

This study included 36 men and 23 women. The mean age at diagnosis was 61.4 (33–80) years in men and 58.5 (32–77) years in women. 72.9% of the patients had an ECOG 0, and none had an ECOG ≥ 2.

As for the histological typification, adenocarcinomas were NOS type in 23 patients (40%), signet ring type in 17 patients (29%), tubular carcinomas in 16 patients (27%) and mucinous carcinomas in 3 patients (5%). The histological degree, according to the WHO, was grade 2 in 29%, grade 3 in 56% and grade 4 in 5% of the patients; this information was not available for one patient (Table 1). Twenty-eight patients (53.8%) presented a stage IIB, followed by stage IIIA (17.3%) and stage IIA (13.4%) (Table 2). The initial clinical stage could not be determined in seven patients. At the time of diagnosis, 50 patients presented lymph node involvement.

**Figure 1.**
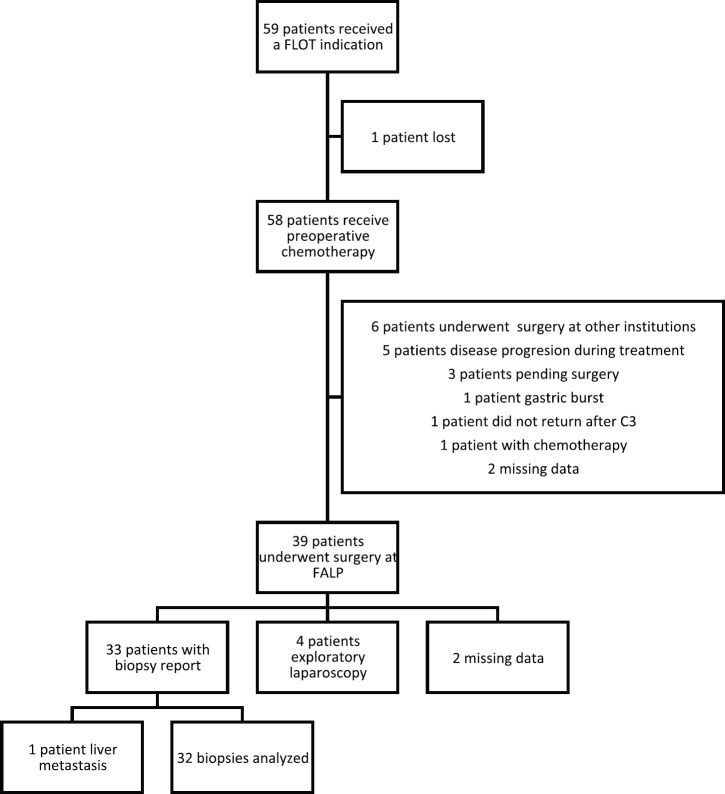
Trial profile. C3: cycle 3, FALP: Instituto oncologico Arturo Lopez Perez, FLOT: fluoruracil, leucovorin, oxaliplatin and docetaxel.

Multidisciplinary oncology team prescribed complete neoadjuvant treatment with FLOT regimen to 59 patients. Fifty-eight patients received any preoperative cycles (Figure 1). The average number of cycles received was 6.41 (2–8 cycles). Forty-two patients (72.4%) received more than four cycles of chemotherapy, and 31 patients (53.4%) received eight cycles of preoperative therapy. Thirty-two patients (55.2%) required a dose reduction of the chemotherapy.

Major adverse event grade 3 or grade 4 occurred in 38 patients (67.8%). No one patient presented adverse event grade 5. Neutropenia was the most frequent complication, and occurred in 21 patients, neutropenia febrile occurred in three patients, while seven patients presented severe gastrointestinal reactions, such as diarrhoea or mucositis, six patients presented fatigue grade 3, five patients presented hyperemesis grade 3 and three patients presented deep vein thrombosis. Severe sepsis occurred in two patients; the original foci were abdomen and lung, respectively. One patient presented a gastric burst during the third cycle of chemotherapy and required surgery. There were no deaths related to therapy.

One patient was still receiving chemotherapy during the analysis of the study. Thirty-nine patients underwent surgery at FALP, and six patients did so at other institutions. Three patients were pending surgery. The data were not available for two patients. One patient presented a severe complication with a gastric burst, and one patient did not return after the third cycle of chemotherapy.

Five patients suffered progression of the disease during chemotherapy. The disease progression of these patients was evaluated by imaging and they did not underwent surgery.

Of the 39 patients that received surgery at our institution, 30 underwent total gastrectomy with D2 dissection. Four patients were subjected to a subtotal gastrectomy. Four patients only had an exploratory laparoscopy because they presented peritoneal carcinomatosis at surgery. Surgery protocol was not available in one patient (Figure 1). Other interventions performed in patients underwent gastrectomy with D2 resections were oophorectomy (one patient), resection of adrenal gland and pillar of the diaphragm (one patient), hyperthermic intraperitoneal chemotherapy (one patient), resection of a liver nodule (two patients) and a pancreatectomy (two patients). The biopsies of all these resections and interventions did not present malignant tumours.

The average number of days between the end of the chemotherapy and the surgery was 49.6 (13–201) days. The complications after surgery were a sepsis of unknown origin in one patient and haematemesis and pneumonia from aspiration in another patient. There were no deaths related to the surgical procedures during the first 30 days of follow-up.

A total of 33 biopsy reports from the patients underwent surgery were obtained. All specimens were adenocarcinomas. The number of lymph nodes of the samples examined was 42.9 (22–59) ganglia, and the average of positive lymph nodes was 5.38 (0–38).

As for the pathologic response observed in only biopsies reported, 6 patients (18.2%) presented a complete pathologic response, and 13 patients (39.4%) had no lymph node involvement and 1 patient presented liver metastasis (Table 3).

At the time of analysis, with a median follow-up time 20.57 months, 21 patients had died, 9 patients had undergone total gastrectomy with D2 dissection, and 1 patient was subjected to partial gastrectomy (Table 4).

The median OS was 21.3 months. 82% and 38% of the patients were alive after 1 and 2 years, respectively.

**Figure 2.**
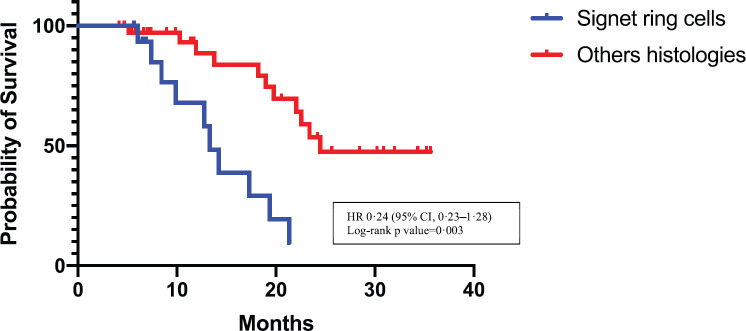
Kaplan–Meier estimates of OS in the signet ring cells group versus the others histologies group. HR = hazard ratio. CI = confidence interval.

Patients with a histology of signet ring carcinoma cells had a shorter survival than those having other histologies: 13.32 months versus 24.46 months, *p* = 0.0003 (95% confidence interval (CI), 0.23 to 1.28), hazard ratio (HR) 0.24 (95% CI, 0.08 to 0.74), respectively (Figure 2).

## Discussion

To the author’s knowledge, this is the first retrospective study to evaluate the administration of the chemotherapeutic FLOT scheme entirely as a preoperative treatment in patients with advanced gastric cancer. The pathologic responses and toxicities were evaluated after the administration of this chemotherapy.

The traditional FLOT scheme comprises four cycles before surgery and an additional four cycles after surgery. This study evaluates the administration of the eight cycles of chemotherapy before surgery with the purpose of achieving higher complete response rates without compromising the safety of the patients.

In comparison to the original studies [5, 6], the demographic characteristics of age are somewhat similar in both studies, and the proportion of women was higher in the present study. The percentage of patients with histology of signet ring adenocarcinoma was similar in both studies. However, there was a higher proportion of patients with cT3, cT4 and N+ in our study, indicating a more advanced presurgical stage in our patients. Another comparison was that only one patient underwent diagnostic laparoscopy before initiating the chemotherapy in comparison to 39% patients of the original study, that fact could imply a sub-staging of the disease in our group of patients before the treatment.

The percentage of patients that received the total number of eight cycles was slightly higher in our study, 53.4% versus 47% in the original study. We were not able to obtain the accumulated doses that they received, but our patients presented a modified dose in 55% of the cases, more significant than the 46% patients of the original studies [5, 6]. The continuity of the treatment and the higher number of cycles could explain this modification of doses. It was not possible to obtain information about the number of patients who used colony-stimulating factors.

Severe adverse events were more frequent in this review, reaching 67.8% in comparison to 41% in the studies [5, 6], probably due to more gastrointestinal events such as mucositis and diarrhoea than recorded in other studies. The most frequent complication was neutropenia, values of which were lower than those observed in studies of the FLOT scheme, probably owing to less monitoring and less use of stimulating factors. Other complications, such as fatigue and infections, were similar to those registered in previous studies. There was a gastric burst after the third cycle of chemotherapy did not record in other studies.

The histopathologic findings after treatment showed that the percentage of patients that reached a T1 or lower tumoural stage was similar to those obtained in earlier studies [6, 7]. However, the proportion of patients with N0 was less in our study.

The proportion of patients who reached a complete pathologic regression was 18% in our report compared to 16% in the previous FLOT study [6]. The value is almost similar; however, this percentage may be underestimated in our study because of variables such as patients with a more advanced clinical stage and the lesser use of staging techniques such as diagnostic laparoscopy. The number of cycles administered could influence the percentage of pathological response; however, a clinical trial with a similar population to pivotal FLOT studies and adequate follow-up should be necessary to determine it.

As in other studies [8], the survival is influenced by the histological type of the disease, being worse in patients carrying signet ring cells than in those with other histologies (HR 0.2423; 95%, 0.07970 to 0.7368).

This study had a small sample of patients, and a follow-up was too limited, which did not allow us to determine the final impact of the therapy on patient survival. However, many other studies have shown that patient survival is higher when the rate of pathological complete response to presurgical chemotherapy is higher.

## Conclusion

In conclusion, total neoadjuvant with FLOT chemotherapy, entirely administered before surgery, presents an adequate safety profile, a similar rate of pathologic regression and a slightly higher rate of completing treatment to report in perioperative FLOT regimen studies. Nevertheless, our group of patients showed more advanced stages of the disease, and probably they were under staging due to lack of exploratory laparoscopy. These factors and an adequate median follow-up could influence the results of the report. A prospective clinical study with suitable diagnostic, staging tools and an adequate follow-up may prove the effectiveness of total neoadjuvant chemotherapy.

## Funding

This work did not receive funding for the study design, the collection, analysis or interpretation of the data, or the preparation of the manuscript.

## List of abbreviations

5-FU, 5-fluorouracil; C3, Cycle 3; D2, Extended lymph node dissection, entailing removal of nodes along the hepatic, left gastric, celiac and splenic arteries, as well as those in the splenic hilum (stations 1 to 12a); ECF, Epirubicin, cisplatin, 5-FU; ECOG, Eastern Cooperative Oncology Group; ECX, Epirubicin, cisplatin, capecitabine; FALP, Instituto Oncologico Arturo Lopez Perez; FLOT, 5-FU, leucovorin, oxaliplatin, docetaxel; IV, Intravenous; OS, Overall survival; pCR, Pathological complete remission.

## Figures and Tables

**Table 1.** Baseline characteristics of population.

Age (years)	All	61.4 (33–80)
	<60	29 (49%)
	60–69	18 (31%)
	>70	12 (20%)
Sex		
	Male	36 (61%)
	Female	23 (39%)
ECOG performance status		
	0	43 (72.9%)
	1	16 (27.1%)
	2	0 (0%)
Albumin (g/dL)	All	3.7 (1.6–4.9)
Haemoglobin (g/dL)	All	12.1 (7.4–15.5)
BMI (kg/m^2^)	All	25.2 (15.4–39.5)
	Male	25.8
	Female	24.3
Histological classification		
	NOS	23 (40%)
	Signet ring	17 (29%)
	Tubular	16 (27%)
	Mucinous	3 (5%)
	Papillary	0 (0%)
	Uncommon variants	0 (0%)
Histological degree (WHO)		
	G1	0 (0%)
	G2	17 (29%)
	G3	38 (66%)
	G4	3 (5%)
	Missing data	1

ECOG, Eastern Cooperative Oncology Group; G1, Grade 1; G2, Grade2; G3, Grade3; G4, Grade4; kg, Kilograms, m^2^, Meter square, NOS, Not otherwise specified; WHO, World Health Organization

**Table 2.** Pathologic stage at initial diagnosis (according to TNM Classification of Malignant Tumours of the UICC, 7th edition).

	*N* (52)
IA	0 (0%)
IB	1 (1.9%)
IIA	7 (13.5%)
IIB	28 (53.8%)
IIIA	9 (17.3%)
IIIB	4 (7.7%)
IIIC	1 (1.9%)
IV	2 (3.8%)

Clinical tumour stage	*N* = 59
cT1	1 (1.7%)
cT2	4 (6.8%)
cT3	47 (79.7%)
cT4	6 (10.2%)
cTx	1 (1.7%)

**Table 3.** Pathologic response after preoperative chemotherapy (according to TNM Classification of Malignant Tumours of the UICC, 7th edition).

	*N* (33)
Complete response	6 (18.2%)
IA	1 (3.0%)
IB	4 (12.1%)
IIA	6 (18.2%)
IIB	4 (12.1%)
IIIA	5 (15.2%)
IIIB	2 (6.0%)
IIIC	4 (12.1%)
IV	1 (3.0%)

Tumour stage (ypT)	*N* = 33
T1	8 (24.2%)
T2	8 (24.2%)
T3	12 (36.4%)
T4	5 (15.2%)

Tumour stage (ypN)	*N* = 33
N0	13 (39.4%)
N1	9 (27.3%)
N2	5 (15.2%)
N3	6 (18.2%)

**Table 4.** Number of patients died according surgical procedures.

Procedures	Number of death
Total gastrectomy and D2 dissection	9
Partial gastrectomy	1
Exploratory laparoscopy	3
Not surgery	3
Data missing	5
Total	21
